# Characterization of Children with Intellectual Disabilities and Relevance of Mushroom *Hericium* Biomass Supplement to Neurocognitive Behavior

**DOI:** 10.3390/nu18020248

**Published:** 2026-01-13

**Authors:** Plamen Dimitrov, Alexandra Petrova, Victoria Bell, Tito Fernandes

**Affiliations:** 1 Department of Psychiatry and Medical Psychology, Faculty of Medicine, Medical University of Varna, 9002 Varna, Bulgaria; 2 Medical Center Plamak, ul. Musala 6, 9000 Varna, Bulgaria; 3 Faculty of Pharmacy, University of Coimbra, Polo das Ciências da Saúde, Azinhaga de Santa Comba, 3000-548 Coimbra, Portugal; 4 LAQV-REQUIMTE, Portuguese Research Centre for Sustainable Chemistry, Rua D. Manuel II, P.O. Box 55142, 4051-401 Porto, Portugal; 5 CIISA, FMV, University of Lisbon, Avenida da Universidade Técnica, 1300-477 Lisboa, Portugal

**Keywords:** *Hericium erinaceus biomass*, bioactive compounds, encephalogram, autism spectrum disorder, neurocognitive disabilities

## Abstract

**Background:** The interplay between neuronutrition, physical activity, and mental health for enhancing brain resilience to stress and overall human health is widely recognized. The use of brain mapping via quantitative-EEG (qEEG) comparative analysis enables researchers to identify deviations or abnormalities and track the changes in neurological patterns when a targeted drug or specific nutrition is administered over time. High-functioning mild-to-borderline intellectual disorders (MBID) and autism spectrum disorder (ASD) constitute leading global public health challenges due to their high prevalence, chronicity, and profound cognitive and functional impact. **Objective:** The objectives of the present study were twofold: first, to characterize an extremely vulnerable group of children with functioning autism symptoms, disclosing their overall pattern of cognitive abilities and areas of difficulty, and second, to investigate the relevance of the effects of a mushroom (*Hericium erinaceus*) biomass dietary supplement on improvement on neurocognitive behavior. **Methods**: This study used qEEG to compare raw data with a normative database to track the changes in neurological brain patterns in 147 children with high-functioning autistic attributes when mushroom *H. erinaceus* biomass supplement was consumed over 6 and 12 months. **Conclusions:** *H. erinaceus* biomass in children with pervasive developmental disorders significantly improved the maturation of the CNS after 6 to 12 months of oral use, decreased the dominant slow-wave activity, and converted slow-wave activity to optimal beta1 frequency. Therefore, despite the lack of randomization, blinding, and risk of bias, due to a limited number of observations, it may be concluded that the *H. erinaceus* biomass may generate a complex effect on the deficits of the autism spectrum when applied to high-functioning MBID children, representing a safe and effective adjunctive strategy for supporting neurodevelopment in children.

## 1. Introduction

The plethora of terms defining conditions such as autistic spectrum disorder that involve limitations in cognitive skills that affect daily functioning and quality of life has evolved over several decades and needs a clearer definition [1]. This is particularly true for ‘high-functioning’ autism in children. This term is not an official medical expression, but it reflects the fact that a child functions relatively well in society, developing a series of coping mechanisms and compensatory strategies, increasing the difficulty of diagnosis [2].

Autism spectrum disorder (ASD) is not automatically considered an intellectual disability, although the two can sometimes occur together. Autism is a neurodevelopmental condition that affects how a person communicates, interacts socially, and processes sensory information. Intellectual disability, on the other hand, involves limitations in intellectual functioning and daily living skills, such as problem-solving, reasoning, and self-care. Around 30 to 70% of people with autism also have an intellectual disability. This means that, while autism and intellectual disability can overlap, they are separate diagnoses with different criteria [3].

In summary, ASD is related to social and behavioral characteristics, while intellectual disability (including mild-to-borderline intellectual disorders, or MBID) is related to cognitive functioning; they are two different diagnostic categories that can be present in the same person. Thus, when it comes to discussions of primary/idiopathic versus secondary autism, considering the role of intellectual disability in diagnosis and phenotyping is critical. In order to decide between ASD features versus MBID as a primary outcome of a trial, the research team must be able to adequately assess both and understand which is more prominent. Because many of the social communication deficits that define ASD represent a failure to acquire developmentally expected skills, these same deficits are expected to occur to some extent in all individuals with intellectual disability [4].

Globally, 1–3% of the population has an intellectual disability, but some remain undiagnosed [5]. Deficits in cognitive and adaptive functioning are the hallmarks of intellectual disability and should be identified during developmental years (childhood through adolescence) [6]. Unprecedented progress in understanding how the brain develops, and in particular, phenomenal changes in both its circuitry and neurochemistry, has been made and is a developing field of neuroscience [7]. While brain development and intelligence are closely linked, brain structure and function play a significant role in cognitive abilities [8].

The fetal brain’s formation begins during the early phases of pregnancy. During gestation, an interface of reciprocal neurobiological changes is induced, since molecular communication between mother and fetus is constantly active and persists even after the fetus starts to synthesize its hormones in late gestation [9]. Although the circulation of the fetus is separated from that of the mother, the trans-placental passage is the source of energy, nutrition, and oxygen through some neurotransmitters (e.g., serotonin, dopamine, and norepinephrine/epinephrine) in the placenta [10]. Throughout the fetal period, the brain undergoes significant growth, and dynamic complex connections are formed as cells multiply and organize; this process can be influenced by both genetics and environmental (nutritional) factors [11]. 

The placenta is crucial for fetal neuronutrition, acting as a dynamic gatekeeper that transports essential nutrients (glucose, amino acids, fatty acids, minerals like iron, zinc, iodine) and synthesizes vital signaling molecules (neurotransmitters like serotonin, dopamine; hormones like IGF-1, thyroid hormones) to support fetal brain development.

An overwhelming series of events at birth, and the months of postnatal experiences and stimulations, does shape the emerging neural circuits. The diverse effects of maternal nutrients intake or status during pregnancy on neurodevelopmental outcomes in children have been studied through neuroimaging [4]. In particular, for brain–function relationships, nutrition is a critical and readily modifiable influence that can profoundly impact early brain maturation. The interplay between neuronutrition, physical activity, and mental health for enhancing brain resilience to stress and overall human health has received significant attention [12].

Autism spectrum disorder (ASD) is a complex neurodevelopmental disorder characterized by atypical behaviors with two core pathological manifestations: deficits in social interaction and communication, and repetitive behaviors [13]. Many of these same factors are also associated with neurodegenerative conditions such as Alzheimer’s disease (AD) and Parkinson’s disease (PD) [14].

ASD is marked by persistent challenges in stimming/self-stimulation and stereotyped movements, diagnosed across a spectrum of severity (Levels 1–3 in DSM-5), with “high-functioning” individuals (often Level 1) showing milder symptoms, distinguished by sufficient language/intellectual skills to mask deficits, leading to challenges like social isolation but potential for independent living, unlike lower-functioning individuals needing significant support [15]. 

In 2025, the APA—American Psychiatric Association—released the DSM-5-TR, a text revision to the DSM-5, which included a clarification to the autism diagnostic criteria. Clinicians use the DSM-5 to evaluate a patient’s symptoms and other criteria to arrive at a diagnosis. It serves as a common language for mental health professionals and is used in a variety of settings, such as clinics, schools, and courtrooms [16]. 

Various micronutrients are used to prevent and treat neurodegeneration and neuropsychiatric disorders; these ‘nootropic’ micronutrients have an impact on redox metabolic homeostasis under physiological and pathophysiological conditions [17]. Nootropic foods are those that may enhance cognitive function, such as memory, focus, and creativity [18,19].

Treatment of autism symptoms usually involves complementary therapies and psychotropic medication. However, despite their benefits, these drugs also have adverse effects [20]. 

### 1.1. Hericium Erinaceus Biomass

**Figure 1:**
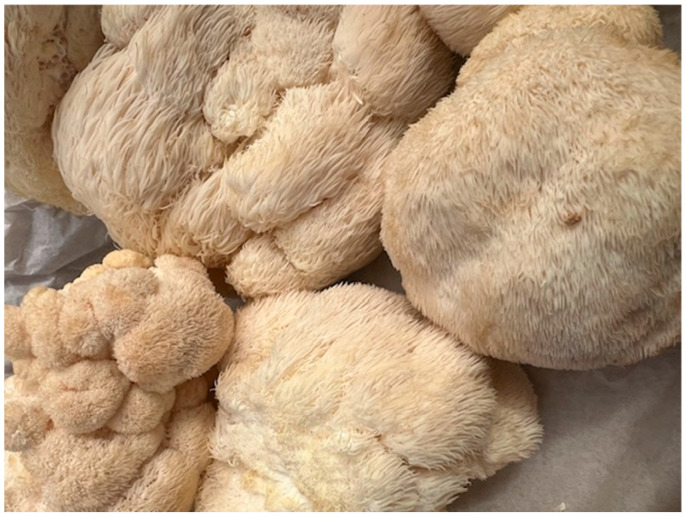
Fresh *Hericium erinaceus* (Bull.) Pers. Lion’s Mane mushroom. Kingdom: Fungi; Phylum: Basidiomycetes; Class: Agaromycetes: Order: Russulales; Family: Hericiaceae; Genus: Hericium; Species: Hericium erinaceus. Authors’ photo.

Mushrooms are fruiting bodies of macroscopic filamentous saprophytic fungi that grow above the ground, presently being used as functional foods [21]. Mushroom dietary supplementation has been used for decades, and *H. erinaceus* (Figure 1) has been the subject of considerable characterization of its bioactive compounds [22] and evaluation in different human health conditions [23,24,25].

*Hericium* represents an attractive natural source for developing novel medicines and functional foods, based on the identification of its active ingredients and metabolites. Also, particularly, *H. erinaceus* primordium contains a high amount of amino acid ergothioneine, the “longevity vitamin”, which has potent antioxidant with anti-inflammatory effects and is produced in nature only by certain fungi and a few microorganisms [26]. 

The *H. erinaceus* biomass (fundamentally different from a *H. erinaceus* extract, which is refined and concentrated) used in all studies mentioned in this article was supplied by MRL—Mycology Research Laboratories Ltd. (Luton, UK). This biomass includes primordia (early fruiting body) and the whole mushroom. Cultivation includes rendering a substrate aseptic, via an autoclave, and inoculating the substrate with a specific strain of *H. erinaceus*.

### 1.2. Hericium Glucan Constituents. Influence on Gut Microbiota

Besides enzymes, the *H. erinaceus* biomass contains both β-glucans and α-glucans, which support the immune system through their immunostimulatory and immunomodulatory properties [27]. In addition, the structure of the biomass protects the constituents from premature proteolytic degradation in the stomach, allowing for a release in the upper intestine [22,28,29].

The vast microbial genetic reservoir within the gut enables the gut microbiota to establish a mutualistic relationship with the host, performing critical functions such as fermenting dietary fibers to produce short-chain fatty acids (SCFAs), synthesizing vitamins (e.g., B12 and K), metabolizing xenobiotics, and competitively excluding pathogens [30].

Dysbiosis, or an instability in gut bacteria, is often observed in individuals with autism, and this imbalance can affect SCFA production. Modulating and optimizing the gut microbiota and increasing SCFA production, especially butyrate, has been shown to be a potential therapeutic approach for autism [31,32].

### 1.3. Increase in Anti-Inflammatory Mediator Lipoxin A4

Lipoxin A4 (LXA4) is a metabolic product of arachidonic acid and is considered an endogenous stop signal for inflammation [33]. A preclinical study was conducted with male Sprague–Dowley rats, determining that supplementation with *H. erinaceus* biomass (given orally at the dose of 200 mg/kg for 90 days) increased Lipoxin A4 in both the body and the brain at the end of the three months [34,35]. This increase was accompanied by increases in the anti-inflammatory vitagenes: heme oxygenase-1 (HO-1), inducible heat shock (Hsp-70), and thiorexdoxin (TrX) in the body and brain in the same period. The supplementation was an equivalent human dose of 3 g per day given orally. These findings suggest that activation of LXA4 signaling and modulation of stress responsive vitagene proteins could serve as a potential therapeutic target for related inflammation and neurodegenerative damage [34,35].

### 1.4. Polyphenols Reducing ROS Levels

The high polyphenolic composition of *H. erinaceus* biomass is responsible for its strong antioxidant properties [36]. Natural polyphenols have been found to have some protective effects against neurodegenerative and neurodevelopmental disorders, which are attributed to a variety of biological properties, particularly antioxidant, immunomodulatory, and anti-inflammatory effects [37,38,39]. These findings provide compelling evidence suggesting that *H. erinaceus* biomass has neuroprotective and health-promoting effects due to redox modulation [22].

*H. erinaceus* terpenoids, like hericenones and erinacines, exert powerful redox effects primarily through antioxidant and anti-inflammatory actions, reducing ROS, boosting antioxidant enzymes (e.g., catalase, glutathione, heme oxygenase-1), and modulating signaling pathways (like Nrf2, NF-κB) to protect neurons, enhance nerve growth, and combat oxidative stress and inflammation linked to neurodegenerative diseases [40].

### 1.5. Disruption of Inflammation

The production of pro-inflammatory cytokines and oxidant molecules produced during an inflammatory response can be reduced by *H. erinaceus* through the action of its bioactive elements (erinacines, hericenones, and polysaccharides), which are able to modulate key inflammatory pathways [41,42,43]. 

### 1.6. Crossing the Blood–Brain Barrier and qEEG

The compounds hericenones (found in the fruiting body) and erinacines (found in the mycelia) of *H. erinaceus* have been shown to pass through the blood–brain barrier and are thus able to exert their neuroprotective and cognitive-enhancing effects within the brain [44].

The local electrical network activity at a given locus within the brain in the presence of drugs can be viewed as an electropharmacogram; this depicts changes in electric activity within specific frequency ranges (e.g., delta, theta, alpha, beta, and gamma) that occur after drug or dietary supplement administration, creating a “fingerprint” or spectral pattern unique to that treatment. This technique helps researchers understand mechanisms of action and potential therapeutic applications [45,46].

Different brain waves are associated with different states and include: Delta (0.5–4 Hz): Deep sleep, healing; excess linked to brain injury/learning issues. Theta (4–8 Hz): Drowsiness, creativity, memory; excess linked to ADHD, anxiety, poor focus. Alpha (8–13 Hz): Relaxed, alert, internal focus; excess linked to anxiety/depression, low alpha to stress/insomnia. Beta (13–30 Hz): Active thinking, focus, alertness; excess linked to stress, anxiety, irritability; low beta to ADHD/depression. Gamma (30–100 Hz): High-level cognition, learning, binding senses; excess linked to stress, high arousal [47].

Electroencephalography (EEG) recordings during administration of a compound may reveal neurophysiological effects at the circuit level whose patterns are specific to a given compound or are specifically shared by a representative psychopharmacological class of compounds [46,48]. Using pharmaco-EEG analysis, rats (n = 9) were supplemented with *H. erinaceus* biomass to confirm whether any ingredients could pass the blood–brain barrier and alter the electric activity of the central nervous system. It was confirmed that *H. erinaceus* biomass could cross the blood–brain barrier [49] and may serve as a promising candidate for the development of novel neuroregenerative therapies [50].

*H. erinaceus* biomass has demonstrated molecular therapeutic action [41]. The use of qEEG comparative analysis seeks to determine the circuit mechanisms that outline the patterns of its neurophysiological effects via pattern comparison with pharmaceutical drugs [51]. With qEEG analysis, researchers noted that a mycological preparation of *H. erinaceus* hyphal powder induced a pattern of frequency changes consisting of a statistically significant attenuation of delta, theta, alpha2, and beta1 spectral power, but not alpha1 power, in all rat brain regions during the first 2 h after administration [49].

The EEG signature of *H. erinaceus* biomass, as determined by discriminant analysis, indicated potential calming, analgesic, antidepressant, and cognitive-enhancing activities with the dose of 150 mg/kg body weight used in this study, which may translate to a human dose of 15 mg/kg body weight, or 1050 mg in a 70 kg adult, within the daily dose range of 1–3 g [49].

The objective of the present study was twofold: first, to characterize an extremely vulnerable group of children with functioning Autism Spectrum Disorder (ASD), disclosing their overall pattern of cognitive abilities and areas of difficulty, and second, to investigate the relevance of the effects of a mushroom (*Hericium erinaceus*) biomass dietary supplement on eventual improvement on neurocognitive behavior.

## 2. Materials and Methods

### 2.1. Participants

The present study involved 147 children aged from 3 to 6 years from different regions of Bulgaria. The Experimental Group (EG) included 57 children (37 boys, 20 girls) with ASD or Asperger’s Syndrome (AS) who met the criteria of “high functioning” autistic syndrome patients—see Table 1 for distribution. The Control Group (CG) included 90 children (60 boys, 30 girls) with neurotypical development.

#### 2.1.1. Inclusion Criteria for Control Group (CG)

Children from the control group had never been diagnosed with any neurodevelopmental disorders or serious illness, regardless of nature, requiring a hospital stay of more than 5 days. They showed normal behavior in the childcare facility they attended, and they had normal development and average success in school.

#### 2.1.2. Inclusion Criteria for the Experimental Group (EG)

All children had a diagnosis of ICD-10 code F84.0 Autism (includes significant language delays and intellectual disabilities) or F84.5 Asperger’s Syndrome (characterized by social and communication difficulties without a delay in language or intellectual development). Both are classified under the broader category of Autism Spectrum Disorder (ASD) in the ICD-10 system. At the time of this study, they were not undergoing any other drug therapy. No co-morbidity from other diseases, such as epilepsy, childhood schizophrenia, severe mental retardation (IQ below 30), cerebral palsy, hydrocephalus, or genetic disease had been registered.

#### 2.1.3. Study Timeline

This study was conducted over 12 months (May 2023–May 2024).

- Baseline (Month 0): qEEG recordings were taken for all participants (CG and EG).
- 6-Month Intervention (Month 6): EG participants underwent repeated qEEG analysis after 6 months of daily supplementation with *H. erinaceus* biomass (designated as EG-6).
- 12-Month Intervention (Month 12): A final qEEG assessment was performed after 12 months of continuous supplementation (EG-12).

All 57 participants in EG-6 continued the treatment to 12 months and formed group EG-12. No one withdrew (due to incompliance, side effects, or other reasons) until the end of the study. The CG children were assessed only at baseline, serving as a static normative comparison. This longitudinal design allows for detection of both short-term neurophysiological effects (after 6 months) and sustained or cumulative changes (after 12 months).

### 2.2. Hericium Erinaceus Biomass Preparation

The mushroom *H. erinaceus* is found practically everywhere, and its safety has been determined [52]. However, its bioactivity varies depending on the environment in which it grows and processing methods. To avoid these variances, an established proprietary strain with quick and hostile colonization was employed. The proprietary strain comprised the full mushroom biomass, not extract, which includes primordia (early fruit body) cultivated under ISO 22000:2023 certification. The 500 mg tablets were produced under food safety certification in the Netherlands.

The optimal adult dosage (3 g/day) was determined based on previous clinical trials in patients with Mild Cognitive Impairment [46] and further supported by preclinical investigations [13,35,53]. In the present study, pediatric dosing was as follows, in accordance with the manufacturer’s recommendations:

Dosing regimen: Children aged 3–4 years: 2 × 500 mg tablets daily (total 1.0 g/day). Children aged 5–6 years: 2 × 2 tablets daily (total 2.0 g/day).

### 2.3. Methods

The Medical Center Plamak/Пламък in Varna, Bulgaria, where the study was conducted, does not have an Ethics Committee established in accordance with international standards. Nevertheless, the highest ethical standards were maintained to protect participants’ rights and welfare, in collaboration with two major University Hospitals in Bulgaria, namely St. Marina University Hospital and Medical University both in Varna. The latter produced the necessary formal Ethical Committee Approval prior to trial initiation. An Ethical Certificate by a specialized Committee is attached to the submission. These included principles like respect for persons, confidentiality, beneficence, and justice, which require researchers to obtain informed consent, protect participants from harm, and ensure fair selection. 

When evaluating an individual with MBID for a potential diagnosis of ASD, it is necessary to be aware of the child’s cognitive ability (based on IQ or developmental test scores) and parents’ information, and to understand any sources or clinical manifestations other than cognitive ability that may have influenced those scores.

Written informed consent was obtained from all parents prior to participation. They were fully informed about the non-toxic nature of the mushroom supplement, the dosing regimen based on previously published scientific data, and the assurance of data protection and confidentiality. Approval for the trial was granted for a 12-month period.

Following screening based on the inclusion and exclusion criteria, parents received detailed information brochures. All legal guardians confirmed voluntary participation by signing informed consent forms. Anonymity, confidentiality, and the exclusive scientific use of collected data were guaranteed. Each parent completed a health questionnaire covering medical history and possible comorbidities that could exclude the child from participation.

EEG recordings were performed using the ELMIKO EEG DigiTrack CFM 4-channel system (manufactured by ELMIKO BIOSIGNALS sp. z o.o., Warsaw, Poland, officially registered in 2008 by the Medicines Control Commission, Republic of Bulgaria), designed for long-term monitoring of brain activity.

The internationally standardized 10–20 system was used for electrode placement, ensuring comparability and reproducibility across subjects and laboratories [54,55].

Data collection was performed at baseline (Month 0), Month 6, and Month 12. For data analysis, internationally standardized frequency ranges were applied (Table 2).

## 3. Results

The results were processed with the Microsoft Office Excel program and SPSS statistical package. One-way analysis of variance (ANOVA) was performed for statistical processing of the measured absolute and relative EEG amplitudes.

The indicators effect strength “F” refers to the F-statistic from an ANOVA, and effect size “η” (etha) refers to eta-squared (η2), a common measure of effect size used in conjunction with ANOVA (72). The F-statistic in an ANOVA test is the ratio of the variance between groups to the variance within groups, calculated as F = mean square between groups divided by mean square within groups. A high F-statistic indicates a large variance between groups. Unlike the F-statistic, which only indicates whether an effect is statistically significant, *η*2 provides information on the practical significance or size of the effect.

F and effect size “η” were both calculated in order to determine how significant were the differences between the average values of the CG children and the EG children after 6 months of oral use of *H. erinaceus* (EG-6) and the same cohort of participants after 12 months with *H. erinaceus* (EG-12).

The values of the indicator were ranked according to the currently widespread classification, where 0.1 is considered a weak effect, 0.24 is medium, 0.37 is greater than typical, and 0.45 and above are much stronger, though these guidelines can vary by field [56].

The relative distribution of amplitudes for individual frequencies (delta, theta, alpha1, alpha1, beta1, and beta2) between the studied groups CG, EG-6, and EG-12 was analyzed. When comparing the three groups—CG, EG-6 and EG-12—a statistically significant difference was found for delta activity with a factor F = 64.06 and the effect size η = 0.57 between the three groups and a slight difference in the relative values of the amplitudes between groups EG-6 and EG-12. Theta activity (F = 13.65 and effect size η = 0.31) displayed a special distribution in which there was a weak statistical difference between the CG and EG-6 groups but a significantly larger difference between the CG and EG-12 groups, as well as between the EG-6 and EG-12 groups. The difference in absolute values of alpha1 activity (F = 53.52; η = 0.54) is significant for all correlations among groups. For alpha2 activity, we have the same distribution (F = 53.12; η = 0.54). Beta1 presents a weaker difference (F = 26.77; η = 0.41), with a significant difference between the CG and EG-6 and EG-12 groups. In the beta2 spectrum, a statistically significant difference F = 33.55; and η = 0.45 is found, with significant differences between the three groups (Table 3 and Table 4). In an ANOVA, the between-group variance measures differences among group means (due to treatment + chance), while the within-group variance (error) measures random spread within each group; the F-ratio compares these.

**Figure 2:**
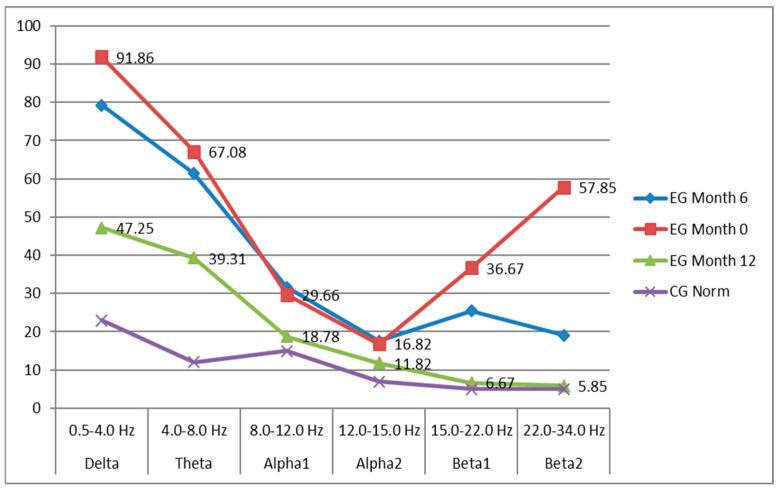
Graphic summary of data from statistical analysis.

The results in percentages for the distribution of delta, theta, alpha1, alpha2, beta1, and beta2 waves are presented in the following graph (Figure 2).

The graph shows spectral EEG power distribution across frequency bands in group EG measured at baseline (Month 0), after 6 months, and after 12 months, compared with group CG (CG Norm)**.** The graph demonstrates a progressive normalization of EEG activity in the experimental group over 12 months, moving from severe dysregulation (excessive delta/theta and beta2) toward a more physiological spectral distribution, approaching control values.

1. Baseline (EG Month 0, red line). At the starting point, qEEG analysis revealed the following patterns:
  - Control Group (CG): Children with neurotypical development displayed age-appropriate qEEG features, including dominant posterior alpha activity (8–12 Hz), balanced theta/alpha ratio, and relatively low-amplitude delta activity, reflecting normal cortical maturation.
  - Experimental Group (EG): Children with ASD/Asperger’s showed marked deviations, consistent with previous literature [57]:
    ▪ Increased theta activity (4–8 Hz), particularly frontally and centrally, suggestive of immature cortical networks and reduced attentional stability.
    ▪ Elevated delta activity (0.5–4 Hz), often reflecting functional disconnection and reduced cortical efficiency.
    ▪ Reduced alpha power (8–12 Hz), indicative of weaker resting-state synchronization.
    ▪ Variability in beta activity (15–22 Hz), with some children showing hyperarousal (excess beta) and others hypoactivation (reduced beta).

These findings confirmed that the EG started from a baseline of dysregulated cortical oscillations, typical for high-functioning ASD/Asperger’s.

2. After 6 Months (EG Month 6, blue line) in the 57 children:
  - Theta power showed a reduction in the frontal and central regions (approx. 12–15% decrease in relative power compared to baseline).
  - Alpha activity demonstrated partial normalization, with increased posterior alpha power, supporting improvements in cortical synchronization and attention regulation.
  - Delta activity showed a modest but statistically significant reduction, indicating better cortical activation.
  - Beta power demonstrated stabilization: children with initial excess beta showed normalization, while those with reduced beta displayed a mild increase.

Clinically, several parents reported improved attention span, better language responsiveness, and reduced irritability in their children, consistent with neurophysiological improvements.

3. After 12 Months (EG Month 12, green line) in the same 57 participants, improvements were more pronounced and stable:
  ▪ Theta/alpha ratio approached values seen in neurotypical controls, suggesting better balance between cortical excitation and inhibition.
  ▪ Alpha coherence (especially inter-hemispheric posterior alpha) increased, a marker of improved integrative processing and functional connectivity.
  ▪ Delta power decreased further, aligning more closely with control norms.
  ▪ Beta1 activity increased moderately in frontal regions, reflecting enhanced executive control and working memory.

Overall, the qEEG profiles of the EG children shifted significantly toward the normative range of the CG, suggesting that *H. erinaceus* promotes cortical maturation and functional reorganization over time. Substantial shifts toward normalization were identified: Delta reduced to 47.25%, much closer to CG (23%); theta and alpha1 moved toward normative values; beta1 and beta2 dropped to near-control levels (6.67% and 5.85%, respectively). These results indicate progressive maturation and stabilization of EEG rhythms.

For more detailed evaluation of child development in the tested groups, the DAYC-2—Developmental Assessment of Young Children [58] was performed to assess cognitive development in the participants, supplemented with *H. erinaceus*. DAYC-2 is a standardized tool widely used in developmental psychology, pediatrics, child neurology, and special education. It is designed to identify developmental delays in young children and to provide a structured profile of their developmental functioning. It is intended to assess global development and to screen for possible developmental delays. The tool is norm-referenced, allowing comparison with age-appropriate expectations. The DAYC-2 is a developmental screening and diagnostic tool assessing five domains of early childhood development (communication, adaptive behavior, cognitive, physical, and social-emotional). It supports early identification of developmental delays and planning of individualized interventions.

**Figure 3:**
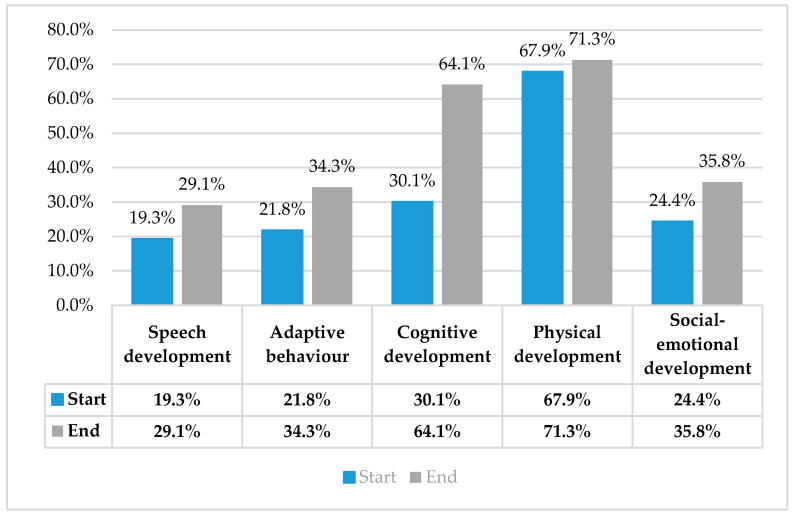
The chart compares developmental domains at two time points: Start (blue bars) and End (grey bars). Domains assessed: Speech, Adaptive behavior, Cognitive development, Physical development, Social–emotional development. Since the standard score for the DAYC-2 is 100, the y-axis shows percentages (%), representing performance or achievement levels.

The results are presented graphically below (Figure 3). The standard score for the DAYC-2 is 100; scores between 90–110% are considered average. A score between 70–78% is two standard deviations below the mean.

### Key Observations by Domain

*Speech development*: Starts at 19.3% and ends at 29.1%. Presents moderate improvement (+10 percentage points). Suggests gradual but positive change in expressive/receptive language abilities. *Adaptive behavior*: Starts at 21.8% and ends at 34.3%. Reveals clear improvement (+12.5 pp). Indicates better ability to cope with daily tasks, routines, and environmental demands. *Cognitive development*: Starts at 30.1% and ends at 64.1%. Very strong improvement (+34 pp). This is the most significant gain, suggesting effective interventions in learning, problem solving, and executive functioning. *Physical development*: Starts at 67.9% and ends at 71.3%. Already high at baseline; only small increase (+3.4 pp). Indicates that motor development was relatively well-preserved from the start, leaving less room for visible growth. *Social–emotional development*: Starts at 24.4% and ends at 35.8%, shows moderate increase (+11.4 pp). Reflects improvement in interaction, emotional regulation, and social integration.

Raw scores may be converted to standard scores and percentiles for each of the five domains. A score falling at or below the 7th percentile (which corresponds to a standard score of 70, or 2 standard deviations below the mean) often marks a significant delay, warranting intervention based on standard cutoffs.

Overall Interpretation:

1. All domains improved between baseline start and end, indicating that the treatment with *H. erinaceus* biomass had a positive global effect on child development.
2. Cognitive development showed the largest relative gain, suggesting strong responsiveness to structured cognitive stimulation.
3. Speech, adaptive behavior, and social–emotional domains improved moderately, but their progress is still behind cognitive and physical growth; these areas may require more targeted interventions.
4. Physical development remained the highest across both points, indicating that motor skills were less affected and less dependent on intervention compared to other domains.

The results demonstrate broad developmental progress, with especially marked improvement in cognitive abilities, while speech and social–emotional skills still represent developmental challenges. This pattern suggests that future focus should be placed on language and social–emotional interventions, as cognitive and physical growth progress more strongly.

## 4. Discussion

In its biological evolution, the CNS is designed for maximum storage of energy, in the form of glucose and oxygen, to support its high metabolic demands. The impaired arrangement of neuronal circuits in neurodevelopmental disorders is expressed in an increased energy inefficiency in maintaining basal activity, and the increased amplitudes are an expression of functional immaturity of the CNS [59]. The use of EEG-based cognitive biomarkers in improving the understanding, diagnosis, monitoring, and treatment of these conditions has been established [60]. Indeed, the EEG-derived markers can reflect neurocognitive dysfunction and inform personalized and scalable mental health interventions.

Beyond animal models, mushroom-based nutritional supplements have demonstrated potential benefits for human health, including applications in ASD and ADHD. Their mechanisms are thought to involve immune modulation, rebalancing of gut microbiota, and attenuation of neuroinflammatory processes—all increasingly recognized as important in the pathophysiology of neurodevelopmental disorders [61].

It was originally thought that important constituents of *H. erinaceus* biomass could not transverse the blood–brain barrier [23]. However, profiling this supplement by an EEG using spectral field power in conscious freely moving rats, determined that the mushroom biomass contains elements that are bioavailable and can cross the blood–brain barrier, having calming, analgesic, antidepressant effects, and cognitive-enhancing activities [49]. The same authors also showed that oral administration of the *H. erinaceus* biomass preparation (150 mg/kg body weight) resulted in a statistically significant attenuation of spectral delta and theta power in the hippocampus and reticular formation. Theta and alpha2 spectral power were statistically significantly attenuated in all brain areas. Also, beta1 spectral power was significantly attenuated in all brain regions [49].

Nutritional mushroom supplements have shown great potential for improving several health issues, and they have been also investigated in the treatment of autism and attention-deficit/hyperactivity disorder (ADHD) regarding the link between immune dysfunction and behavioral traits [62]. They have also been shown to have a positive effect in rebalancing and regulating the gut microbiota and the immune system while helping to reduce neuroinflammation [63,64].

Relative and absolute amplitude values for the children from the present experimental group’s EG-6 and EG-12 were significantly higher than from the children of the control group CG, which may be explained by the expression of multiple biological factors. One of the explanations for the increased amplitude is that it is a sign of “immaturity” in the functioning of the children’s CNS [65].

In the present study, when comparing the amplitudes between the three groups, a significant difference in beta2 activity emerges, which is an expression of distress and mental overstrain of nervous activity. We believe that through beta2 activity the autistic brain tries to compensate the insufficiency of long brain connections by over activating local micro connections. Remarkable results were detected after 12 months of oral use of *H. erinaceus* biomass in the EG improving the beta2 activity, confirming other recent data [41].

Another important finding was the difference in theta activity. This frequency (4–7 Hz) is not a direct criterion for the integrity of the sensory system in the human nervous system, but it is significant in cognitive and memory processes [66]. This result is an expression of a disturbed neurophysiological substrate in children with ASD. When comparing the CG and the summarized values of the EG, the significance of the differences in theta activity is higher than that of the beta-2 frequency. If we consider only this difference, we can assume that dysfunction in sensory integrity is of greater importance in the pathophysiology of autism [67].

In some of the studied children with Asperger’s syndrome and autism, there was a significant disproportion in theta activity compared with the control group. Theta activity is a direct expression of the processes in the primary sensory processing of incoming stimuli, both from the external environment and from the somatic sensorium [68].

Dysfunction in this activity is directly related to sensory disintegration, which is observed in many of the children on the spectrum. Apparently, these children are clumsy, with relatively late walking and unstable gait and balance. Often, these are children with strong sensitivity to even the weakest stimuli (generally sensory, auditory, and visual) or under conditions of strong suppression of sensory processing [69]. These children manifest insensitivity and a high threshold for any type of irritation, to the point of not reacting to pain. Disorders in theta activity are often associated with slow-type hyperactive behavior, although the ratio of theta/beta has been challenged as a reliable diagnostic tool [70,71].

We have identified statistically significant differences in the relative signal strength in the brain waves of the studied spectrum when compared the three groups separately, between the Control (CG) and the Experimental Group (EG), and between the EG-6 and EG-12 groups. First, significant differences between the three groups separately are found in the delta activity. Delta brainwave frequency (0.5–4 Hz) is basic, the slowest type, and a fundamental frequency generated by the deep structures of the brain, such as the brainstem and the reticular formation located in it. Delta activity sets the basic rhythm to which all adjacent functionally active structural neural networks are tuned [72,73].

This vital function of the reticular formation, which regulates consciousness and arousal, is called in the specialized literature the Ascending Reticular Activating System (ARAS), a complex network of interconnected brain nuclei and pathways linking the brainstem and the cerebral cortex, which together regulate waking, alertness, attention, and dreaming states through changes in brain wave frequency and amplitude [74].

Delta activity, a hallmark of the EEG, is an expression of the maturity of the CNS, reflecting the ongoing rhythmic electrical activity of the brain. The more dominant slow-wave activity in the waking state links to more “immature” brain activity [75]. The autistic brain is functionally slow-wave dominant, with an inability to regulate the associated processes that are generated in the sub-cortex to reach the cortical fields [76]. The delta rhythm is considered to activate all ascending structures and modulate all brain functions [75].

In the absence of good connectivity between cortical structures, the CNS autonomously generates high beta activity to compensate for the deficit of the basic delta frequency. There are hypotheses that this high beta activity (as we noted in this study) is a consequence of the established larger amount of short intragyrus interneuronal connections and the deficit of distant cortical-subcortical and intracortical pathways, such as fronto-thalamic, fronto-parietal, thalamo-cortical tracts [77].

The improvement in delta activity achieved following the administration of *H. erinaceus* biomass is substantial: a slight improvement between the groups’ EG-0 and EG-6, and a larger significant improvement between EG-0 and EG-12.

Specific statistically significant differences in the relative amplitude values of the alpha spectrum can occur due to various factors, including differences in age, sex, personality traits, and even the presence of certain neurological conditions like ADHD. The absolute values of the alpha waves were close in value between the control and experimental groups.

Individuals from the CG had relatively high absolute alpha values compared with other frequencies. Children with ASD present generally increased values of all amplitudes, including alpha activity. Against the background of the distribution of the remaining waves in the spectrum and the conducted relative analysis of the percentage ratio between them, we concluded that there is a deficit of alpha activity in the experimental group. This is a significant distinguishing marker in the functional deficit of people with autism suggesting the potential of alpha connectivity as biomarkers for ASD as also recently found by other authors [78].

The deficit in alpha activity between 8 and 12 Hz and at the specific frequency between 12 and 15 Hz in children with autism can be explained by the following arguments. Neural networks with the participation of mirror neurons and von Economo neurons (VENs, also known as spindle cells) [79] are crucial for social cognition, action understanding, and emotional processing, and it has been established that these networks and systems oscillate functionally exclusively in the alpha range [80]. These neural networks are responsible for important mental qualities, information processing such as analyzing new situations, and representing systems for learning and building new models from the earliest childhood [81]. These are networks of empathic connections with which we become aware of the feelings and mental states of individuals closest to us and subsequently of the people in our environment [82].

These abilities are missing in people suffering from ASD. During the 6- and 12-month treatment with *H. erinaceus*, it was seen that the relative share of alpha activity is maintained or increased throughout the entire period, which contributes to the improvement of the general activity of the CNS. The weaker statistical difference in theta activity between 4 and 8 Hz in CG and EG children may be explained since oscillations in this range are generated mainly by structures of the midbrain, such as the thalamus and hippocampus, with a projection mainly to the frontal cortical areas [83].

The present findings support the profound neuromodulatory actions of *H. erinaceus* biomass on CNS function, particularly in children on the autism spectrum. Twelve months of oral administration in children with high-functioning ASD (F84.0) and Asperger’s syndrome (F84.5), aged 3–6 years, significantly promoted CNS maturation by reducing pathological slow-wave activity and enhancing optimal beta1 activity.

Six and twelve months of supplementation improved sensory–motor functions, as reflected in the modulation of theta activity. These effects involve subcortical structures such as the thalamus and limbic system and regulate the striatal system to enhance coordination of fine motor movements. Twelve months of supplementation also stabilized alpha activity. Alpha deficits are particularly detrimental in ASD, as they are linked to impaired social behavior and communication. Restoration of alpha rhythms contributes to improvements in language development and grammatical competence.

In summary, although *H. erinaceus* affected favorably both EG6 and EG12 groups, there were specific differences, decreasing the dominant slow-wave activity and converting slow-wave activity to optimal beta1 frequency.

Much more fundamental research in the field is needed to definitely prove this claim, but these are probably the main pathophysiological and psychoneurological markers for autism order.

## 5. Limitations and Future Perspectives

Although the present study aimed to characterize qEEG in children with intellectual disabilities and the effects of nutritional mushroom biomass supplement on children with neurodevelopmental disorders and behavioral symptoms, what was obtained was merely a snapshot, and conclusions only point towards a positive direction and not a strict model to follow, at present.

Knowingly, to overcome present bias, there is the need to perform randomized, double-blind, placebo-controlled clinical trials to confirm present tendency of efficacy of *Hericium* biomass, if possible with other multiple aspects taken into consideration. Indeed, the influence of the supplement on modulation of the microbiome, reducing dysbiosis, and enhancing gut barrier integrity, needs investigation.

Although studies have been conducted in the past in animals and in vitro with this mushroom, future studies should investigate biological mechanisms through inflammatory biomarkers, microbiota, and pediatric pharmacokinetics. To avoid chance findings, response moderators must be assessed by identifying variables (like age, gender, severity, or expectations) that may change the strength or direction of the effect, leading to personalized medicine and better intervention design.

Concomitant therapies must be checked for eventual drug interactions with the supplement, affecting medication efficacy, or causing adverse effects, requiring healthcare provider guidance for safe integration, especially with neurologic conditions, to prevent negative outcomes and ensure optimal patient care. Furthermore, towards a personalized proposal, a just one-size-fits-all approach may not be justified as diversity exists. 

A key trend in neuroscience must be taken into consideration by combining EEG, neuroimaging, and behavioral scales to offer a complete picture of brain function, moving beyond just immediate changes to see if neural and behavioral benefits last, which is crucial for effective therapy design and predicting sustainability of outcomes. Integrating clinical, functional, and quality-of-life outcomes is crucial for future advancement regarding the use of the *Hericium* supplement in autism spectrum.

## 6. Conclusions

Medical treatment for autism spectrum is influenced by factors like symptom diversity, complex etiology, and ethical concerns. The use of dietary supplements as an adjuvant therapy appears to be a safe and effective approach to enhance communication, to manage sensory sensitivities, and to develop social independence in these individuals.

Despite the blurred diagnostic boundaries between MBID and ASD, it was demonstrated that daily *H. erinaceus* supplementation in children with symptoms is an interesting hypothesis of neuronutrition, as they showed significant improvements, with the limitations already enhanced.

Better outcomes were specifically observed:

1. A reduction in pathological slow-wave activity (theta, delta).
2. Enhancement of alpha synchronization and interhemispheric coherence.
3. Stabilization and normalization of beta rhythms.
4. Overall cortical electrophysiological maturation, approaching the patterns observed in neurotypical children.

Further research using randomized controlled designs, larger samples, and multimodal outcome measures (behavioral, cognitive, and neuroimaging) is warranted.

The present data indicate that oral administration of *H. erinaceus* biomass (150 mg/kg body weight) attenuated spectral delta and theta power in both the hippocampus and reticular formation. Moreover, theta and alpha2 power were reduced across all brain regions, with a concurrent attenuation of beta1 activity. Jointly, these findings suggest widespread neuromodulatory effects on cortical and subcortical oscillatory activity, requiring further exploratory trials.

The present outcomes are clinically relevant but not transformative due to sample size and other constraints. This approach still needs improvements towards the development of personalized intervention strategies. Furthermore, no side effects were observed after oral supplementation with *H. erinaceus* biomass, since no dysbiosis or other hindrance was observed.

The present findings, combined with neuroanatomical studies of individuals on the autism spectrum, highlight some abnormalities in frequency ratios, spectral power, coherence, and hemispheric asymmetry. In addition to the limitations mentioned, there are critical methodological and clinical issues that must be considered in both data interpretation and the design of future studies.

## Figures and Tables

**Table 1:** Breakdown of 147 study subjects by CG and EG.

	Ages 3 to 6 Years	Male n (%)	Female n (%)
A	Control Group (CG)	60 (66.7%)	30 (33.3%)
B	Supplemented/Experimental Group (EG)		
	Asperger Syndrome (F84.5)	16 (28.1%)	9 (15.8%)
	Autistic Spectrum Disorder (F84.0)	21 (36.8%)	11 (19.3%)
		37 (64.9%)	20 (35.1%)

**Table 2:** Brain wave spectra (Hz). Each wave corresponds to different states of brain activity.

Delta	Theta	Alpha1	Alpha2	Beta1	Beta2
0.5–4.0	4.0–8.0	8.0–12.0	12.0–15.0	15.0–22.0	22.0–34.0

**Table 3:** ANOVA analysis of the significance of the differences (F factor) for the relative values of the amplitudes of the individual frequencies between groups “CG”, “EG-6”, and “EG-12” (*p* < 0.001).

		Sum of Squares	Mean Square	F	Sig.
Delta	Between Groups	0.529	0.264	64.056	
	Within Groups	1.085	0.004		*p* < 0.001
Theta	Between Groups	0.045	0.022	13.650	*p* < 0.001
	Within Groups	0.33	0.002		
Alpha1	Between Groups	0.195	0.097	53.516	*p* < 0.001
	Within Groups	0.479	0.002		
Alpha2	Between Groups	0.063	0.031	53.124	*p* < 0.001
	Within Groups	0.155	0.001		
Beta1	Between Groups	0.046	0.023	26.774	*p* < 0.001
	Within Groups	0.225	0.001		
Beta2	Between Groups	0.091	0.045	33.547	*p* < 0.001
	Within Groups	0.356	0.001		

**Table 4:** Summary data from statistical analysis of the relative values for Factor F differences between CG/EG-6/EG-12, and between EG-6 and EG-12 on the left and right hemispheres.

	Left Hemisphere		Right Hemisphere	
	CG/EG6/EG12	EG6/EG12	CG/EG6/EG12	EG6/EG12
Delta	64.06	1.94	75.99	5.51
Theta	13.65	26.11	14.38	29.55
Alpha1	53.52	53.62	43.78	41.85
Alpha2	53.12	1.35	46.41	1.54
Beta1	26.74	10.36	32.72	3.28
Beta2	33.55	64.98	25.88	57.70

## Data Availability

The raw datasets generated and statistically analyzed for the present study comprise several appendix with individual records and can be made available from the corresponding author on reasonable request. There is no restriction at all but to be able to present the results in summary.
